# Comparative genomics and functional analysis of niche-specific adaptation in *Pseudomonas putida*

**DOI:** 10.1111/j.1574-6976.2010.00249.x

**Published:** 2010-08-26

**Authors:** Xiao Wu, Sébastien Monchy, Safiyh Taghavi, Wei Zhu, Juan Ramos, Daniel van der Lelie

**Affiliations:** 1 Biology Department, Brookhaven National LaboratoryUpton, NY, USA; 2 Department of Applied Mathematics & Statistics, State University of New YorkStony Brook, NY, USA; 3 EEZ-CSICGranada, Spain

**Keywords:** *Pseudomonas putida*, comparative genomics, W619, KT2440, F1, GB-1

## Abstract

*Pseudomonas putida* is a gram-negative rod-shaped gammaproteobacterium that is found throughout various environments. Members of the species *P. putida* show a diverse spectrum of metabolic activities, which is indicative of their adaptation to various niches, which includes the ability to live in soils and sediments contaminated with high concentrations of heavy metals and organic contaminants. *Pseudomonas putida* strains are also found as plant growth-promoting rhizospheric and endophytic bacteria. The genome sequences of several *P. putida* species have become available and provide a unique tool to study the specific niche adaptation of the various *P. putida* strains. In this review, we compare the genomes of four *P. putida* strains: the rhizospheric strain KT2440, the endophytic strain W619, the aromatic hydrocarbon-degrading strain F1 and the manganese-oxidizing strain GB-1. Comparative genomics provided a powerful tool to gain new insights into the adaptation of *P. putida* to specific lifestyles and environmental niches, and clearly demonstrated that horizontal gene transfer played a key role in this adaptation process, as many of the niche-specific functions were found to be encoded on clearly defined genomic islands.

## Introduction

The group of the pseudomonads comprises a heterogeneous set of microorganisms that can be isolated from many different niches (Clarke, 1982). They belong to the class *Gammaproteobacteria* and nearly 100 different strains have been described. Within this group of microorganisms, a number of strains have been associated to the species *Pseudomonas putida*, and in this review, we analyzed some of the most characteristic features derived from the analysis of their genome sequences. Bacteria of this species are known for their ability to colonize soils and for their capacity to degrade a wide variety of chemicals, including many natural and man-made compounds.

The rhizospheric bacterium *P. putida* KT2440 was isolated from garden soil in Japan based on its ability to use 3-methylbenzoate (Nakazawa, 2002). Since the publication of its complete 6.18 Mbp genome sequence in 2002, which represented the first sequenced *Pseudomonas* genome and that allowed a comparative analysis of the metabolically versatile *P. putida* KT2440 as a function of this strain's ability to survive in marginal and polluted soils (Nelson *et al.*, 2002), our knowledge of this strain has increased significantly. As a result, *P. putida* KT2440 represents the best-characterized strain from this species, and is considered as the workhorse for *Pseudomonas* research (Timmis, 2002; Dos Santos *et al.*, 2004;). *Pseudomonas putida* KT2440 is a microorganism generally recognized as safe (GRAS certified) for the cloning and expression of foreign genes, and although there is a high level of genome conservation with the pathogenic species *Pseudomonas aeruginosa* (85% of the predicted coding regions are shared), key virulence factors including exotoxin A and type III secretion systems were found to be lacking from its genome (Nelson *et al.*, 2002). Its unusual wealth of determinants for high-affinity nutrient acquisition systems, mono- and di-oxygenases, oxido-reductases, ferredoxins and cytochromes, dehydrogenases, sulfur metabolism proteins, for efflux pumps and glutathione-*S* transfereases and for the extensive array of extracytoplasmatic function sigma factors, regulators and stress response systems constitutes the genomic basis for the exceptional nutritional versatility and opportunism of *P. putida* KT2440. This metabolic diversity was successfully exploited for the experimental design of novel catabolic pathways as well as for its application in the production of high-added value compounds whose synthesis is not coupled to cell survival (Dos Santos *et al.*, 2004). These include the hyperproduction of polymers (such as polyhydroxyalkanoates) (Huijberts *et al.*, 1992; Huijberts & Eggink, 1996;), industrially relevant enzymes, the production of epoxides, substituted catechols, enantiopure alcohols and heterocyclic compounds (Schmid *et al.*, 2001). Biotechnology applications of *P. putida* KT2440 were further facilitated by the genome-scale reconstruction and analysis of metabolic network models (Nogales *et al.*, 2008; Puchalka *et al.*, 2008;). Both models were used successfully to improve the production of polyhydroxyalkanoates from various carbon sources as examples of how central metabolic precursors of a compound of interest not directly coupled to the organism's growth function might be increased via the modification of global flux patterns. Because the species *P. putida* encompasses many strains with a wide range of metabolic features and numerous isolates with unique phenotypes, these models provide an important basic scaffold upon which future models of other *P. putida* strains, specifically addressing important properties for their niche-specific adaptation such as the growth of *P. putida* F1 on aromatic hydrocarbons, can be tailored as a function of strain-specific metabolic pathways.

*Pseudomonas putida* W619 is a plant growth-promoting endophytic bacterium, which was isolated from *Populus trichocarpa*×*deltoides* cv. ‘Hoogvorst,’ and represents one of the most commonly found species among the poplar endophytes isolated so far (Taghavi *et al.*, 2005, 2009). Very similar bacteria were isolated from the poplar rhizosphere, and as endophytes from root and stem material. Like *P. putida* KT2440, strain W619 is an excellent host for the expression of foreign genes. This property was exploited to successfully introduce and express the ability to degrade toluene and trichloroethylene (TCE). *In situ* bioaugmentation with *P. putida* W619-TCE reduced TCE evapotranspiration by 90% under field conditions. This encouraging result, which represents one of the few examples of successful bioaugmentation, was achieved after the establishment and enrichment of *P. putida* W619-TCE as a poplar root endophyte and by further horizontal gene transfer of TCE metabolic activity to members of the poplars endogenous endophytic population (Weyens *et al.*, 2009). Because *P. putida* W619-TCE was engineered via horizontal gene transfer, its deliberate release is not restricted under European genetically modified organisms regulations.

*Pseudomonas putida* F1 was isolated from a polluted creek in Urbana, IL, by enrichment culture with ethylbenzene as the sole source of carbon and energy. F1 is considered a reference strain for the degradation and growth of *P. putida* on aromatic hydrocarbons, including benzene, toluene, ethylbenzene and *p*-cymene. Mutants of strain F1 that are capable of growing on *n*-propylbenzene, *n*-butylbenzene, isopropylbenzene and biphenyl as the sole carbon sources are easily obtained (Choi *et al.*, 2003). In addition to aromatic hydrocarbons, the broad substrate toluene dioxygenase of strain F1 can oxidize TCE, indole, nitrotoluenes, chlorobenzenes, chlorophenols and many other substituted aromatic compounds (Spain & Gibson, 1988; Wackett & Gibson, 1988; Spain *et al.*, 1989;). Although *P. putida* F1 cannot use TCE as a source of carbon and energy, it is capable of degrading and detoxifying TCE in the presence of an additional carbon source. The ability of *P. putida* F1 to degrade benzene, toluene and ethylbenzene has a direct bearing on the development of strategies for dealing with environmental pollution.

*Pseudomonas putida* GB-1 was isolated from fresh water and characterized as a robust manganese (Mn^2+^) oxidizer (Rosson & Nealson, 1982). When supplied with Mn^2+^, the cells deposit manganese oxide outside the outer membrane during the early stationary growth phase, a process that is enzymatically catalyzed (Okazaki *et al.*, 1997). This process was further studied at both the genetic and the physiological level, making GB-1, along with the closely related strain MnB1, the model organism for studies of Mn(II) oxidization (Okazaki *et al.*, 1997; Caspi *et al.*, 1998; de Vrind *et al.*, 1998; Brouwers *et al.*, 1999; Villalobos *et al.*, 2003;).

This review focuses on the diversity and adaptation of four *P. putida* strains (KT2440, W619, F1 and GB-1) in terms of their respective environments from a genome point of view, and provides further insights into their potential applications for the bioremediation of contaminated environments or as plant growth-promoting bacteria.

## Comparative genomics of *P. putida*

### Structure and general features of the *P. putida* genomes

**Fig. 1:**
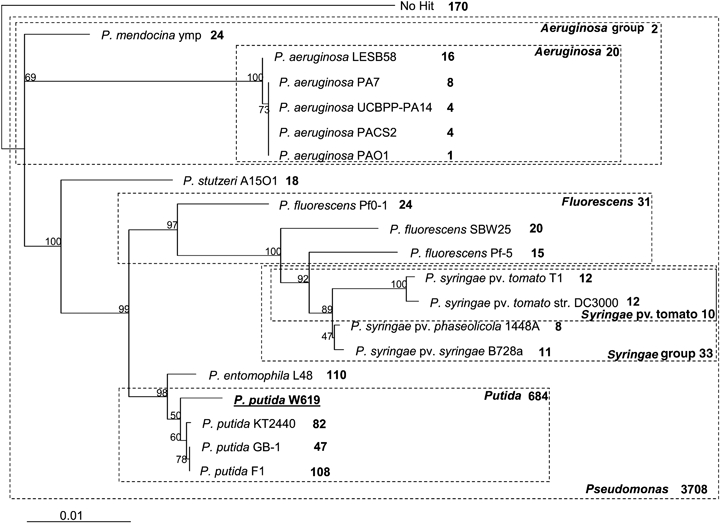
Phylogenetic relationship between members of the genus *Pseudomonas*. 16S rRNA gene comparison was used to build the phylogenetic tree for those members of the genus *Pseudomonas* with publicly available genome sequences. The numbers at nodes represent the bootstrap values (1000 replicates), and the numbers in bold correspond to the number of coding sequences (CDS) preferentially shared by W619 and the corresponding organisms (with *E* value of 10^−5^). The numbers of CDS equally found in two or more organisms are indicated for this subset of organisms (boxed with dotted lines) that correspond to a group of taxa/phylum according to the NCBI taxonomy. Whole-genome comparison for shared CDS was based on megan (Huson *et al.*, 2007).

A whole-genome comparison using megan (Huson *et al.*, 2007) between *P. putida* W619 and the publicly available genome sequences of members of the genus *Pseudomonas* reveals the presence of 3708 shared coding sequences (CDS) (Fig. 1). An additional 684 CDS are shared between W619 and the genomes of the other three *P. putida* strains, plus 82, 47 and 108 CDS are uniquely shared between W619 and strains KT2440, GB-1 and F1, respectively. Among the *Pseudomonas* sp. outside the species *P. putida, Pseudomonas entomophila* L48 is the closest relative, sharing 110 additional genes with W619. Because this review focuses on the genomic analyses of representative strains of *P. putida, P. entomophila* L48 has not been included in this comparison. It should also be noticed that W619 contains 170 CDS that have no hit (at *E* value of <10^−5^) to any CDS present among the sequenced *Pseudomonas* genomes, which could indicate that these genes potentially originate from organisms outside of the genus *Pseudomonas*.

**Fig. 2:**
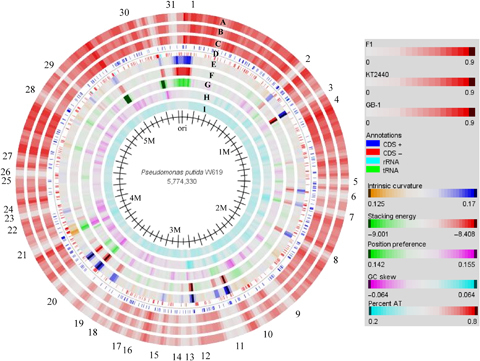
Genome atlas for the chromosome of *Pseudomonas putida* W619. The numbers outside the atlas indicate the locations of the predicted genomic islands on the W619 chromosome. Details of these genomic islands are provided in Table S1. From the outside to the inside the circles represent the three blast atlases for chromosome comparisons with *P. putida* strains F1 (a), KT2440 (b) and GB-1 (c), respectively, followed by an overview of specific W619 chromosome properties: (d) annotations for coding sequences on the +and – strand, rRNA and tRNA genes; (e) intrinsic curvature; (f) stacking energy; (g) position preference; (h) GC skew; and (i) percent AT. The explanation of the color codes is presented in the figure legend. The atlas was generated using the GeneWiz browser 0.91 (http://www.cbs.dtu.dk/services/gwBrowser/).

**Fig. 3:**
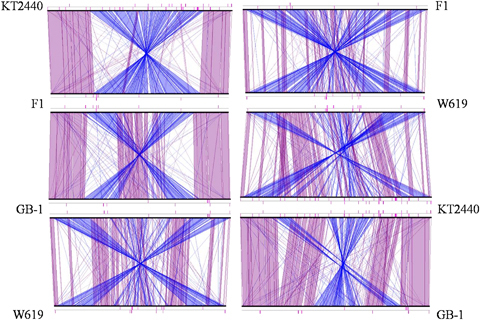
Comparative syntheny line plots showing the orthologous relations among *Pseudomonas putida* W619, F1, GB-1 and KT2440. The synteny regions are displayed with strand conservation (in purple) and stand inversion (in blue). The pink bars represent the positions of transposable elements. The ‘Conserved Synteny LinePlot’ tool in MaGe was used to generate this figure (Vallenet *et al.*, 2006). The corresponding RefSeq synteny statistics are provided in Table S2.

The *P. putida* W619 genome was manually annotated using the MaGe annotation system (Vallenet *et al.*, 2006) (http://www.genoscope.cns.fr/agc/website/), and compared with the well-analyzed KT2440 and automatically annotated GB-1 and F1 genomes. The general genome features of the four *P. putida* strains are summarized in Table 1. Strains W619, F1 and GB-1 are predicted to encode 5471 CDSs with a coding density of 89%, 5300 CDSs with a coding density of 90% and 5417 CDSs with a coding density of 90%, respectively. These findings are comparable to the 5420 CDSs predicted for KT2440, with a coding percentage of 86%. The four strains share many general genome features. For example, all possess a single circular chromosome that displays a clear GC skew transition. Their chromosomal replication origin has the typical organization for *P. putida*, which is distinct from the enteric-type origins (Yee & Smith, 1990; Weigel *et al.*, 1997;). The *ori*C site locates between the *rpm*H and the *dna*A genes and contains conserved DnaA-binding boxes (TTATCCACA). Figure 2 shows the Genome Atlas of *P. putida* W619 (Hallin *et al.*, 2009; Ussery *et al.*, 2009;) with customized blast lanes added for F1, KT2440 and GB-1. The outer three lanes show genes of W619 shared with the other three *P. putida* strains. The numbers around the chromosomal atlas indicate the locations of the predicted genomic islands in W619 (see details in Supporting Information, Table S1). These regions are generally characterized by lack of synteny, along with a high intrinsic curvature and stacking energy, which is indicative of their higher recombination activity as compared with other chromosomal regions that seem to constitute the *P. putida* core genome. The putative orthologous relations and synteny group arrangements of the four genomes are illustrated in the comparative syntheny line plots (Fig. 3) and by the syntheny statistics (Table S2). Based on the comparative synteny line plots (Fig. 3), *P. putida* W619 has considerable inverted alignments on both sides of the chromosomal replication origin as compared with the other three *P. putida* strains. This indicates that the W619 genome might have undergone a major DNA rearrangement that involved swapping two DNA segments that are flanking the chromosomal replication origin. Members of the genus *Pseudomonas* are characterized by their ability to grow on defined minimal media using a huge variety of organic compounds as energy and carbon sources. The biosynthetic pathways for proteinogenic amino acids and vitamins have been solved recently for *P. putida* KT2440, and this information is well conserved in the other analyzed strains of this species (Molina-Henares *et al.*, 2009, 2010). Using the COGs functional categories in the NCBI database, the *P. putida* strains were compared with the *Gammaproteobacteria* and total bacteria, and were found to share a similar distribution for most COG classes (Table S3) (Tatusov *et al.*, 2000). However, all four *P. putida* strains displayed a higher percentage of genes putatively coding for signal transduction mechanisms (T), pointing to the presence of sophisticated regulatory networks to control gene expression in function of a highly variable environment, and inorganic ion transport and metabolism (P). On the other hand, the *P. putida* genomes were less dense in genes putatively involved in carbohydrate transport and metabolism (G) and replication, recombination and repair (L).

**Table 1:** Comparison of the general genome features of *Pseudomonas putida* strains W619, F1, GB-1 and KT2440

	W619	F1	GB-1	KT2440
Number of bases	5 774 330	5 959 964	6 078 430	6 181 860
Number of CDSs	471	5300	5417	5420
CDSs with predicted function (%)	70.0	73.5	74.9	76.9
CDSs without function with a homolog (%)	25.6	23.6	22.7	19.1
CDSs without function without a homolog (%)	4.4	0.65	0.7	4.0
Pseudogenes	26	49	8	ND
Coding %	89	90	90	86
%GC	61	61	62	62
tRNAs	75	76	74	73
rRNA genes (clusters)	22 (7)	20 (6)	22 (7)	22 (7)
Putative orthologous relations (%)				
W619	100	77	75	75
F1	–	100	80	82
GB-1	–	–	100	77
KT2440	–	–	–	100

The numbers of genome features have been retrieved from the NCBI site (http://www.ncbi.nlm.nih.gov/sites/genome/) and the JGI site (http://img.jgi.doe.gov/cgi-bin/pub/main.cgi). ND, not determined; the absence of pseudogenes in KT2440 is based on information provided by NCBI, which might be out of date. Orthologous relations are predicted using the RefSeq synteny statistic in MaGe platform (Vallenet *et al.*, 2006).

### Mobile elements in *P. putida*

Conserved IS elements between the *P. putida* strains were identified using the IS Finder (http://www-is.biotoul.fr/) database and blast searches with a stringent *E* value threshold (below e^−100^). Complete putative IS elements identified in W619, KT2440, F1 and GB-1 are listed in Table 2A. As can be seen in the table, the KT2440 genome contains 36 IS elements, a very high number especially in comparison with strain F1 (only 2 IS elements of the IS*3* and IS*5* families, respectively), with the majority of the IS elements in KT2440 being present in multicopies. In comparison, strains W619 and GB-1 contain eight and nine IS elements, respectively. The high number of IS elements may be related to the versatile catabolic ability of KT2440, which also requires an increased level of genome plasticity in order to adapt to various environments, this in comparison with the other strains that thrive in more specialized niches. When applying a higher threshold *E* score, several incomplete or truncated remnants of IS elements were identified among the various genomes (Table S4).

**Table 2:** Mobile elements' comparison for the *Pseudomonas putida* strains W619, KT2440, F1 and GB-1

Family/Group	Name	Origin	Length	IR	DR	W619ORF ID (PputW619)	KT2440ORF ID (PP_)	F1ORF ID (Pput_)	GB-1ORF ID (PputGB1_)
*(A) IS elements*									
IS*3*/IS*51*	IS*Ppu22*	*P. putida* GB-1	1232	29/45	3	–	–	0419-0415	1613-16142972-29713805-38065424-5425
IS*4*/IS*4*	IS*Ppu8*	*P. putida* KT2440	1419	18	11	–	1865 19902114 22182522 4318	–	–
IS*5*/IS*5*	IS*Pa16*	*P. aeruginosa*	1192	12	4	3354	–	–	–
IS*5*/IS*5*	IS*1246*	*P. putida* mt-2 (pWWO)	1275	15/16	ND	–	2971	–	–
IS*5*/IS*5*	IS*2000*	*P. aeruginosa* JES	1186	14/17	4	–	–	1356	–
IS*5*/IS*427*	IS*Ps1*	*P. syringae*	>203 (800-1000)	ND	ND	2346-2347	–	–	4818-48174824-4825
IS*30*	IS*1382*	*P. putida* H	1093	35	3	1990	–	–	-
IS*66*	IS*Ppu14*	*P. putida* KT2440	2383	27/33	8	0037-0039 5173-5175	3501-34993964-39663979-39814443-44414439-44375398-5396	–	–
IS*66*	IS*Ppu15*	*P. putida* KT2440	2041	22/28	8	–	0638-06374024-40254092-40914746-4745	–	0521-05221718-17194791-4792
IS*66*	IS*Ppu13*	*P. putida* KT2440	2370	19/22	8	–	3986-39843113-3115	–	–
IS*110*	IS*Ppu9*	*P. putida* KT2440	2043	6/11	2	–	1133 1260 2570 3381 3586 4603 4791	–	–
IS*110*	IS*Ppu10*	*P. putida* KT2440	1314	0	0	–	0526 1653 2134 3502 4599 50505290	–	–
IS*110*/IS*1111*	IS*Ppu11*	*P. putida* KT2440	1348	12	0	–	0334 3498	–	–
IS*256*	IS*Pa27*	*P. aeruginosa*	1361	23/29	7	3315	–	–	–
IS*1182*	IS*Ppu16*	*P. putida* KT2440	1677	15/16	2	3317	1931	–	–
ISL*3*	IS*Ppu12*	*P. putida* pWW0	3372	21/24	ND	2325-2328	–	–	–

IS elements were identified using IS Finder: http://www-is.biotoul.fr/									
IR, the length(s) of the terminal IR(s) in base pairs. A single number refers to two IRs with the same length; DR, the number of target base pairs duplicated on insertion; ND, not determined.									

W619^*^		KT2440			F1			GB-1	
*(B) Phage*									
PputW6191301-1357		PP1536-1584			Pput_3359-3401			PputGB1_1181-1221^a^	
PputW6193919-3971^b^		PP2266-2297			Pput_4096-4150^a^			PputGB1_1720-1762	
PputW6194030-4047^a^		PP3026-3066						PputGB1_3388-3473	
		PP3859-3920						PputGB1_4147-4177^b^	

^*^Phages with the same label inserted in the same position on the genomes of respective strains. The presence of phages was predicted using the prophinder (Lima-Mendez *et al.*, 2008) tool.									

Putative product (gene)^*^			W6 19 ORF ID (PputW619)		F1 ORF ID (Pput_)		KT2440 ORF ID (PP_)	GB-1ORF ID (PputGB1_)	
*(C) Other transposable elements*									
Tn*7* family protein (*tnsA*)			5195^a^		–		5406^g^	–	
Tn*7* family protein (*tnsB*)			5194^a^		–		5405^g^	–	
Tn*7* family protein (*tnsC*)			5193^a^0036		–		5404^g^	–	
Tn*7* family protein (*tnsD*)			5192^a^		–		5407^g^	–	
Tn*4652* cointegrate resolution protein T (*tnpT*)			2672^b^2275^c^		2522^f^		32392982^h^	2650^i^	
Tn*4652* cointegrate resolution protein S (*tnpS*)			2680^b^2276^c^		2535^f^		31812981^h^	2669^i^	
Tn*4652* transposase subunit AB			–		–		2976–2977	–	
Tn*4652* transposase (*tnpA*)			–		–		2964	–	
Tn*4652 tnpA* regulatory protein (*tnpC*)			–		–		2965	–	
Tn*3* transposase(*tnpA*)			2321^d^		–		–	–	
Tn*3* resolvase (*tnpR*)			2322^d^4533		–		–	–	
Resolvase (*tniR*)			2332^e^		–		–	–	
Truncated transposase (*tniA*)			2334^e^		–		–	–	
Tyrosine recombinase (*xerC*)			0242		5139		5230	5291	
Tyrosine recombinase (*xerD*)			4154		4253		1468	1073	

^*^ORFs with the same label are located in the same region on the chromosome and are supposedly part of the same composite transposable element. IS elements were identified using IS Finder: http://www-is.biotoul.fr/

It should also be noted that several of the IS elements were unique to a *P. putida* genome, including seven copies of both IS*Ppu9* and IS*Ppu10*, six copies of IS*Ppu8*, two copies of both IS*Ppu13* and IS*Ppu11* and a copy of IS*1246* found in KT2440, copies of IS*Pa16*, IS*Pa27* and IS*1382* in W619 and a copy of IS*2000* in F1. None of the identified IS elements was present on more than two of the genomes.

IS elements are often associated with resistance and accessory functions that bacteria have acquired via horizontal gene transfer, or with DNA rearrangements in order to affect the expression or the stability of the newly acquired functions (Mahillon & Chandler, 1998). For example in *P. putida* W619, the unique *mhp* aromatic degradation operon was found between one incomplete copy of IS*1182* (PputW619_1976-1977) and the copy of IS*1382* (PputW619_1990), indicating that this operon was acquired via horizontal gene transfer. Also, a mercury resistance operon was found adjacent to the unique copy of IS*Ppu12* (PputW619_2325-2328) (Williams *et al.*, 2002), while in its proximity, other transposable elements were identified, including a putative Tn*3* family transposon (PputW619_2321-2322), a recombinase and a truncated transposase. Therefore, this gene segment might represent a composite mercury resistance transposon in W619, acquired via horizontal gene transfer and absent in *P. putida* F1, KT2440 and GB-1. Additional transposable elements are summarized in Table 2C. Among them, *tnsABCD* from the Tn*7* family was found in association with a gene cluster encoding heavy metal resistance in W619 and KT2440.

The prophinder (Lima-Mendez *et al.*, 2008) tool was used for prophage prediction in the *P. putida* genomes. Prophages were identified in all four genomes (Table 2B), with their numbers ranging between 2 and 4. Several prophages were found to be inserted at the same location among different strains. For example, of the three prophages found in strain W619, one was found at the same location in strain GB-1, while another prophage (PputW619_4030-4047) was found inserted adjacent to a ferredoxin-related gene in both GB-1 (PputGB1_1181-1221) and F1 (Pput_4096-4150). This points to the existence of an insertion hotspot for this particular prophage in the *P. putida* core genome (Manna *et al.*, 2004).

### Genomic islands in *P. putida*

We predicted specific genomic regions for each of the four *P. putida* strains in comparison with the other three strains using the MaGe annotation system (Vallenet *et al.*, 2006). Based on the automatic prediction algorithm, 61, 54 and 66 putative regions were identified in *P. putida* KT2440, F1 and GB-1, respectively; along with the manual annotation of W619, 31 putative genomic islands were identified for *P. putida* W619 (Table S1). Different from 105 predicted genomic islands of KT2440 as described by Weinel *et al.* (2002), a number that was based on the compositional bias of the GC, di- and tetranucleotides of the chromosome itself, the 61 putative genomic islands in KT2440 that we identified in our analysis were predicted based on the lack of synteny against the genomes of the other three *P. putida* strains, GC bias and the existence of mobile genetic elements. For example, genomic island 19 (coordinates 922000–945000) predicted by Weinel *et al.* (2002) was not included in our analysis because this region is conserved in the other three *P. putida* strains. On the other hand, region 31 (coordinates 3121963–3224467) of KT2440, which was not considered as a genomic island by Weinel *et al.* (2002), lacks synteny to the genomes of W619 and GB-1, and carries genes that are involved in sugar transport (PP_2757-PP_2761).

Among the various putative genomic islands, many are suggested to have been acquired via horizontal gene transfer due to the presence of integrases or mobile genetic elements (transposons, IS elements) and an alternative matrix of codon usage compared with the rest of the chromosome. As shown in the following sections, these putative genomic islands have conferred the *P. putida* species with many accessory capabilities, including heavy metal resistance, aromatic compound degradation and stress responses, all of which are highly relevant for the specific niche adaption of *P. putida*.

## Adaption of *P. putida* to a polluted soil environment

### Heavy metal resistances in *P. putida*

Based on its genome sequence, *P. putida* KT2440 was predicted to tolerate various heavy metals (Canovas *et al.*, 2003), and this strain's metal resistance properties were experimentally confirmed. *Pseudomonas putida* W619 strain was originally isolated from the roots of poplar cuttings that were obtained from trees growing on a field site aimed to remediate groundwater contaminated with various compounds, including BTEX compounds (benzene, toluene, ethylbenzene and xylene), TCE and Ni (Taghavi *et al.*, 2005). It was therefore expected that strain W619 also possesses the capacity to deal with heavy metals. Based on the strain's genome sequence, 78 genes were identified that encode proteins putatively involved in heavy metal resistance and homeostasis, a number that exceeds the number of genes identified on the KT2440 genome (Canovas *et al.*, 2003). Furthermore, when comparing the MIC values of strains W619 and KT2440 for various heavy metals, we found that W619 displayed an increased resistance to Cd(II) (MIC values of 0.5 and 0.25 mM for W619 and KT2440, respectively), Cu and Ni (see details below) and similar resistance to Zn(II) (MIC 0.5 mM) and Co(II) (MIC 0.1 mM). We therefore used the W619 genome as the reference to compare the different *P. putida* genomes for functions putatively involved in heavy metal resistance.

**Fig. 4:**
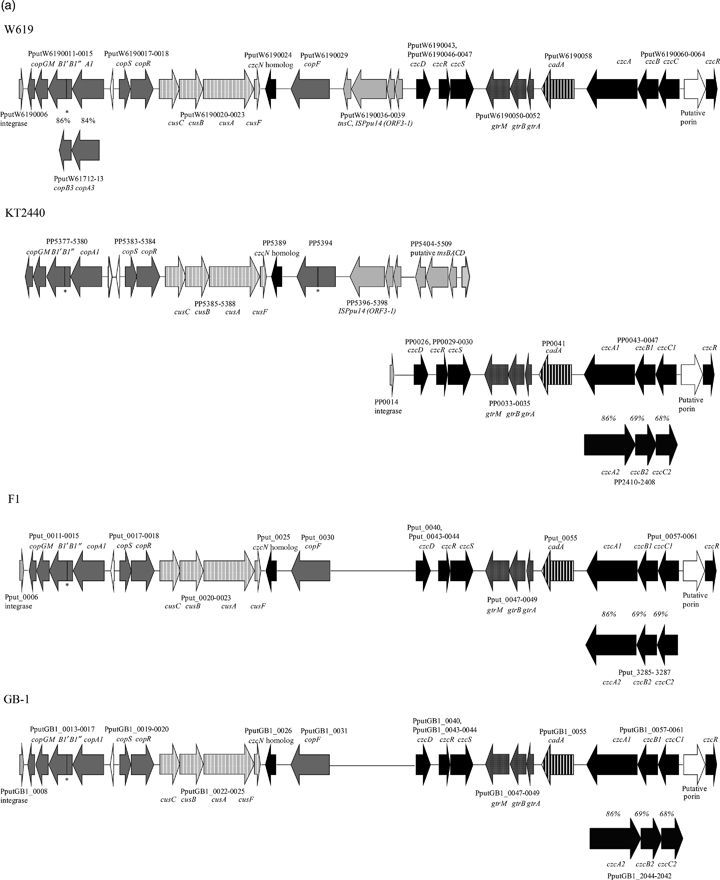

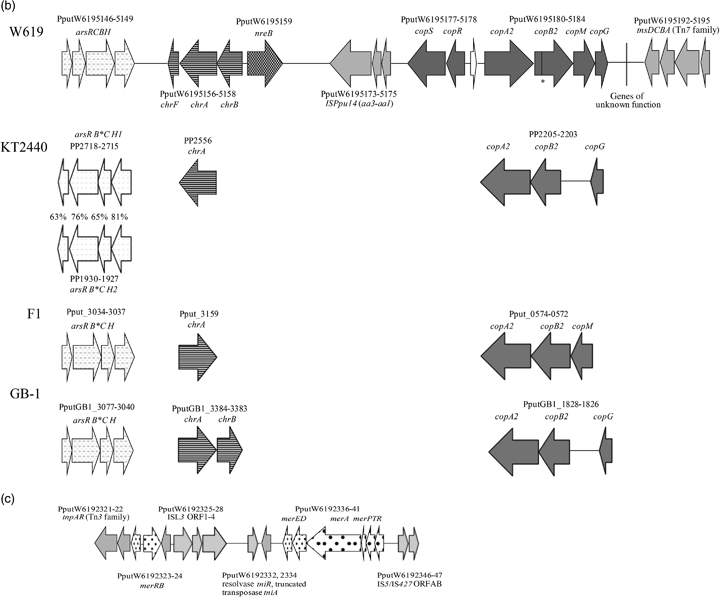
Genetic organization of heavy metal resistance determinants located on the chromosomes of *Pseudomonas putida* W619, KT2440, F1 and GB-1. (a) Genetic organization of heavy metal resistance determinants on the putative genomic island region 1 of *P. putida* W619, and its comparison with the corresponding regions located on the chromosomes of *P. putida* KT2440, F1 and GB-1. PP5394, located on the *P. putida* KT2440 chromosomes, encodes a P-type ATPase (Ag/Cu efflux), which was reported to have a frameshift mutation (http://www.tigr.org). Identities are provided as percentages between two copies of homologous genes. ^*^ The position of a frame shift mutation in comparison with the full-length ORF. (b) Genetic organization of heavy metal resistance determinants on putative genomic island region 31 of *P. putida* W619, and its comparison with the corresponding regions located on the chromosomes of *P. putida* KT2440, F1 and GB-1. The *arsB* gene in *P. putida* W619 and the arsB^*^ genes in *P. putida* KT2440, F1 and GB-1 are from different sources. (c) The mercury resistance region and its flanking mobile genetic elements on the chromosome of the *P. putida* W619 genome. This region is absent in *P. putida* KT2440, F1 and GB-1.

The majority of the *P. putida* W619 heavy metal resistance genes are found on two genomic regions, referred to as region 1 and 31 that are located on opposite sites of the chromosomal origin of replication. A similar organization is observed for F1 and GB-1 and in some way for KT2440. Most of the heavy metal resistance genes located in region 1 (coordinates 7447–75941) are conserved among all four *P. putida* strains (Table 3 and Fig. 4a). On the 5′-end, this region is delimitated by an integrase gene (PputW619_0006). This integrase is also found in the equivalent regions of F1 and GB-1, but is absent in KT2440. Interestingly, region 1 contains three different systems for putative copper resistance: *copRSABMG* (PputW619_0018-11) for periplasmic detoxification, with CopA being a multicopper oxidase (MCO) that is thought to oxidize Cu(I) to Cu(II) in the periplasm (Rensing & Grass, 2003), and CopB being an outer membrane protein (Cha & Cooksey, 1991); a P-type ATPase gene *copF* (PputW619_0029) for the cytoplasmic detoxification of monovalent Cu(I); and the copper/silver resistance operon *cusFcusABC* (PputW619_0023-0020) for the periplasmic detoxification of Cu(I) and Ag(I) via expulsion by the three-component efflux system (*cus*ABC) across the outer membrane. The accessory protein CusF is believed to be a periplasmic copper chaperon delivering Cu(I) to the CusABC complex (Franke *et al.*, 2003). Region 1 also seems to encode for resistance to cadmium, zinc and cobalt based on the presence of the *czcABC* (a three-component efflux system for the periplasmic detoxification of Cd, Zn and Co) and the *czcDRS* gene [including the cation diffusion facilitator (CDF) protein CzcD] clusters with some rearrangement (PputW619_0060-62, 0043, 0046-0047). In addition, a *cadA* gene (PputW619_0058), encoding a CadA P-type ATPase, is present as well as a *gtrABM* (PputW619_0052-0050) locus putatively involved in heavy metal resistance by repairing the membrane, whose integrity may have been altered by the precipitation of heavy metal on the bacterial surface. The clustering of the *czc* efflux system, the membrane maintenance genes *gtrABM* and a putative porin (PputW619_0063) (Fig. 4a) are features that resemble those described for the Cd, Zn and Co resistance cluster (ORF82-106) of the *Cupriavidus metallidurans* CH34 plasmid pMOL30, but with some rearrangements (Monchy *et al.*, 2007). It should also be noted that a *czcN* homolog, described to play a role in the regulation of the *czc* operon (Dong & Mergeay, 1994; Grosse *et al.*, 1999;), is located 18 kb upstream of the *czc* cluster in between the putative *cusF* and *copF* genes.

**Table 3:** Summary and comparison of genes found in *Pseudomonas putida* that might be involved in metal resistance and homeostasis based on genomic analysis

Gene	Metal	W619 (region)*ORF ID PputW619_	F1 ORF ID Pput_	KT2440 ORF ID PP_	GB-1 ORF ID PputGB1_	Product
*copG*	Cu	0011 (1)	0011	5377	0013	Involved in survival in the presence of high bioavailable Cu(II)
*copM*	Cu	0012 (1)	0012	5378	0014	Cytochrome *c* family protein
*copB*′	Cu	0013 (1)	0013	5379	0015	Copper resistance protein B
*copB*″	Cu	0014 (1)	0014	–	0016	Putative CopB (frameshifted)
*copA*	Cu	0015 (1)	0015	5380	0017	Copper resistance protein A
*copR*	Cu	0017 (1)	0017	5383	0019	Transcriptional activator CopR
*copS*	Cu	0018 (1)	0018	5384	0020	Sensor protein CopS
*cusC*	Cu/Ag	0020 (1)	0020	5385	0022	Cu/Ag tricomponent efflux outer membrane porin
*cusB*	Cu/Ag	0021 (1)	0021	5386	0023	Cu/Ag tricomponent efflux membrane fusion protein
*cusA*	Cu/Ag	0022 (1)	0022	5387	0024	Copper transporter, RND family
*cusF*	Cu/Ag	0023 (1)	0023	5388	0025	Periplasmic copper-binding protein
-	?	0024 (1)	0024	5389	0026	*S*-isoprenylcysteine methyltransferase (czcN homolog)
*copF*	Cu	0029 (1)	0030	–	0031	Copper P-type ATPase
*czcD*	Cd/Zn/Co	0043 (1)	0040	0026	0040	Cation Diffusion Facilitator (CDF)
*czcR*	Cd/Zn/Co	0046 (1)	0043	0029	0043	DNA-binding response regulator
*czcS*	Cd/Zn/Co	0047 (1)	0044	0030	0044	Sensory histidine kinase
*gtrM*	–	0050 (1)	0047	0033	0047	Glycosyltransferase (protein *o*-glycosylation)
*gtrB*	–	0051 (1)	0048	0034	0048	Glycosyltransferase (bactoprenol)
*gtrA*	–	0052 (1)	0049	0035	0049	Bactoprenol-linked glucose translocase (Flippase)
*cadA*	Cd/Zn	0058 (1)	0055	0041	0055	Cadmium translocating P-type ATPase
*czcA*	Cd/Zn/Co	0060 (1)	0057	0043	0057	Cobalt/zinc/cadmium efflux RND transporter
*czcB*	Cd/Zn/Co	0061 (1)	0058	0044	0058	Cobalt/zinc/cadmium efflux RND transporter
*czcC*	Cd/Zn/Co	0062 (1)	0059	0045	0059	Cobalt/zinc/cadmium resistance protein
-	Cd/Zn/Co	0063 (1)	0060	0046	0060	Putative porin, OprD family
*czcR*	Cd/Zn/Co	0064 (1)	0061	0047	0061	DNA-binding heavy metal response regulator
*cadR*	Cd/Zn	0325	5013	5140	5193	Transcriptional regulator
*cadA*	Cd/Zn	0326	5012	5139	5192	Cadmium translocating P-type ATPase
*arsC*	As	1207	4072	1645	1247	Arsenate reductase
*cinA*	Cu	1676	3583	2159	1700	Copper-containing azurin-like protein
*cinQ*	Cu	1677	3584	2160	1701	Pre-Q_0_ reductase
*copB*	Cu	1712 (8)	–	–	–	Copper resistance protein B
*copA*	Cu	1713 (8)	–	–	–	Copper resistance protein A
-	Zn	1714 (8)	–	–	–	Cation efflux protein (Putative Zinc transporter ZitB)
*arsB*	As	1715 (8)	–	–	–	Arsenite efflux pump
*merR*	Hg	2323 (11)	–	–	–	Mercuric resistance operon regulatory protein
*merB*	Hg	2324 (11)	–	–	–	Alkylmercury lyase (organomercurial lyase)
*merR*	Hg	2325 (11)	–	–	–	Transcriptional regulator, MerR-family
-	?	2326 (11)	–	–	–	Heavy metal/H+ antiporter, CDF family
*merE*	Hg	2336 (11)	–	–	–	Mercuric resistance protein
*merD*	Hg	2337 (11)	–	–	–	HTH-type transcriptional regulator
*merA*	Hg	2338 (11)	–	–	–	Mercuric Hg(II) reductase
*merP*	Hg	2339 (11)	–	–	–	Mercuric transport protein periplasmic component
*merT*	Hg	2340 (11)	–	–	–	Mercuric transport protein
*merR*	Hg	2341 (11)	–	–	–	Mercuric resistance operon regulatory protein
*nikR*	Ni	3004 (18)	–	3341	–	Putative nickel-responsive regulator
*nikA*	Ni	3005 (18)	–	3342	–	Nickel ABC transporter, periplasmic nickel-binding protein
*nikB*	Ni	3006 (18)	–	3343	–	Nickel transporter permease NikB
*nikC*	Ni	3007 (18)	–	3344	–	Nickel transporter permease NikC
*nikD*	Ni	3008 (18)	–	3345	–	Nickel import ATP-binding protein NikD
*nikE*	Ni	3009 (18)	–	3346	–	Nickel import ATP-binding protein NikE
*chrA*	Cr	3017 (18)	–	–	–	Chromate transporter
*modA*	Mo	3197	1942	3828	3543	Mo ABC transporter, periplasmic Mo-binding protein
*modB*	Mo	3198	1941	3829	3544	Mo ABC transporter, permease protein
*modC*	Mo	3199	1940	3830	3545	Mo ABC transporter, ATP-binding protein
*arsC*	As	4088	4193	1531	1140	Arsenate reductase (glutaredoxin family)
-	Cu	4576	0627	0588	0633	Putative copper-binding protein
-	Cu	4578	0625	0586	0631	Putative copper-translocating P-type ATPase
*merE*	Cu	4579	0624	0585	0630	Transcription regulator heavy metal-dependent MerE family
*znuA*	Zn	5108	0137	0120	0135	Zinc uptake ABC transporter, periplasmic binding protein
*zur*	Zn	5109	0136	0119	0134	Transcriptional repressor of Zn transport system
*znuC*	Zn	5110	0135	0118	0133	Zinc ABC transporter, ATP-binding protein
*znuB*	Zn	5111	0134	0117	0132	Zinc ABC transporter, permease protein
*arsR*	As	5146 (31)	3034	2718/1930	3077	Arsenical resistance operon repressor
*arsC*	As	5147 (31)	3036	2716/1928	3079	Arsenate reductase
*arsB*	As	5148 (31)	3035	2717/1929	3078	Arsenite efflux transporter
*arsH*	As	5149 (31)	3037	2715/1927	3080	Arsenical resistance protein
*chrF*	Cr	5156 (31)	–	–	–	Chromate resistance regulator
*chrA*	Cr	5157 (31)	3159	2556	3384	Chromate transporter
*chrB*	Cr	5158 (31)	–	–	3383	Chromate resistance protein
*nreB*	Ni	5159 (31)	–	–	–	Major facilitator superfamily (nickel efflux family)
-	?	5161 (31)	–	–	–	Putative cation diffusion facilitator (CDF)
*copS*	Cu	5177 (31)	–	–	–	Sensor protein CopS
*copR*	Cu	5178 (31)	–	–	–	Transcriptional activator CopR
*copA*	Cu	5180 (31)	0574	2205	1828	Copper resistance protein A
*copB*″	Cu	5181 (31)	–	–	–	Putative CopB (frameshifted)
*copB*′	Cu	5182 (31)	0573	2204	1827	Copper resistance protein B
*copM*	Cu	5183 (31)	0572	–	–	Cytochrome *c* family protein
*copG*	Cu	5184 (31)	–	2203	1826	Involved in survival in the presence of high bioavailable Cu(II)
*czcC*	Cd/Zn/Co	–	3287	2408	2042	Cobalt/zinc/cadmium resistance protein
*czcB*	Cd/Zn/Co	–	3286	2409	2043	Cobalt/zinc/cadmium efflux RND transporter
*czcA*	Cd/Zn/Co	–	3285	2410	2044	Cobalt/zinc/cadmium efflux RND transporter
*mrdH*	Ni/Cd/Zn	–	–	2968	–	Ni/Cd/Zn resistance-associated protein
*mreA*	?	–	–	2969	–	Metal resistance-associated cytoplasmic protein

* For the reference genome W619, the numbers in brackets indicate the genomic region of the gene, which was omitted if the gene was not located in a genomic region.

?, the metal specificity remains to be determined. The gene annotations and comparisons were obtained using the MaGe annotation platform (Vallenet *et al.*, 2006).

In between *copF* and *czcDRS*, a copy of IS*Ppu14* (PputW619_0037-0039) was found, separating the copper/silver resistance cluster from the cadmium/zinc/cobalt resistance cluster. The region 1 counterparts of F1 and GB-1 seem to be identical in organization with very close synteny to W619, the only difference being the absence of IS*Ppu14*. In the case of KT2440, region 1 is divided into two sections that are separated by the chromosomal replication origin: section 1, containing the copper/silver resistance cluster that ends with a copy of IS*Ppu14*, and section 2, which starts with a putative integrase and contains the cadmium/zinc/cobalt resistance cluster.

Region 31 (coordinates: 5706900–5753604) of *P. putida* W619 is less conserved among the other three *P. putida* strains, and contains genes involved in arsenite/arsenate, chromium, nickel and copper resistances (Fig. 4b). The arsenite/arsenate resistance operon (*arsRCBH*; PputW619_5146-5149) has homologs on the KT2440, F1 and GB-1 genomes, except that their *arsB* genes are different: the gene from W619 is closely related to the *arsB* gene from *Acinetobacter baumannii* AYE, while the *arsB* genes from the other *P. putida* strains are closer to the *Escherichia coli* K12 *arsB*. Still, *P. putida* W619 and KT2440 showed similar growth and resistance to As(III) and As(V) in the range of concentration we tested (both viable in the presence of NaAsO_2_ (0.01 to ∼2 mM) and Na_2_HAsO_4_ (0.001 to ∼5 mM). Besides this *arsRCBH* operon, two additional *arsC* genes encoding the arsenate reductase were present on the *P. putida* chromosomes, as well as an additional copy of *arsRCBH* in KT2440 (PP_1927-1930).

The chromate resistance genes *chrFAB* (PputW619_5156-5158) and the nickel resistance gene *nreB* (PputW619_5159) are located downstream of the *ars* operon, and are incomplete or absent in KT2440, F1 and GB-1. They are followed by a second copy of the copper resistance genes *copSRABMG* (PputW619_5177-5178, 5180-5184), which is flanked by a copy of IS*Ppu14* (PputW619_5173-5175) and by a copy of a Tn*7*-like transposon (*tnsABCD*; PputW619_5195-5192). Remnants of the *cop* operon are found in KT2440, F1 and GB-1. As for region 1, the region 31 *cop* operon seems to be part of a composite mobile element. Although the organization of both *cop* operons is very similar, they showed only 80% protein identity, which rules out a recent duplication.

The *cop* determinant in *Pseudomonas syringae* pv. *tomato* contains six genes (*copABCDRS*) (Mellano & Cooksey, 1988). However, the presence of *copAB* was sufficient to render the cells copper resistant (Mellano & Cooksey, 1988; Cha & Cooksey, 1993;). CopAB are known to be involved in the detoxification and efflux of copper ions from the periplasm (Rensing *et al.*, 2000; Monchy *et al.*, 2006;). In *P. syringae*, the CopA and CopB proteins contain the MXXMXHXXM (MDH) motif repeated several times throughout the sequence. This motif also appears in CopA1 (PputW619_0015), where it is repeated 12 times. However, this repeat is truncated for CopA2 (PputW619_5180) and is only present five times. A similar organization was described for the two *copA* genes in KT2440 (Canovas *et al.*, 2003), having this motif repeated 14 and five times in CopA1_KT2440_ and CopA2_KT2440_, respectively, and was also observed for F1 and GB-1.

Both copies of CopB in W619 contained a frame shift mutation. For instance, in the case of CopB1, if this frame shift was ignored, the resulting complete protein would contain six copies of the MDH motif and 21 histidine residues, which are known to bind copper efficiently. The truncated CopB1 protein, however, only contains three MDH motifs and 13 histidine residues. A similar frame shift was also found in KT2440, F1 and GB-1, but only in one of the two CopB copies. In addition to the two copies of *copSRAB*, an additional *copAB* locus was identified on the W619 genome (PputW619_1712-1713, located in region 8; coordinates: 1875105–1910820), which might make up for the frame shift mutations. In addition, W619 has a *copF* gene putatively encoding a P1-type ATPase, which is located in the inner membrane and involved in Cu(I) efflux from the cytoplasm to the periplasm (Rensing *et al.*, 2000; Mergeay *et al.*, 2003;). The *copF* gene is absent in KT2440, which might explain the higher level of copper resistance for W619 compared with KT2440, with MIC values of 0.5 and 0.1 mM Cu, respectively.

Because *P. putida* W619 was isolated from a nickel-contaminated site, it was no surprise that it showed elevated levels of nickel resistance compared to KT2440, with MIC values of 1 and 0.1 mM, respectively. The only putative nickel resistance protein identified in W619 was a copy of *nreB* (PputW619_5159), located in region 31, which is absent in KT2440, F1 and GB-1. The presence of *nreB* is consistent with the observation that in *A. xylosoxidans* and *E. coli, nreB* expression was sufficient for nickel resistance (Grass *et al.*, 2001). In KT2440, a novel metal resistance determinant *mrdH* (PP_2968) was identified recently, which encodes a protein with a chimeric domain organization from RcnA and CzcB (Haritha *et al.*, 2009). MrdH was found to be involved in nickel, cadmium and zinc resistance. The *mrdH* gene and *mreA* (PP_2969), the latter showing similarity to NreA-like proteins, lack homologs in *P. putida* W619, GB-1 and F1, and are located on genomic island 34 (GI 55 according to Weinel *et al.* 2002) of KT2440. The presumably higher specificity for nickel resistance of *nreB* compared with *mrdH* may explain strain W619's much higher MIC value for Ni(II) as compared to KT2440. It should also be noted that the activities of the mobile genetic elements Tn*4652* (PP_2964-65) and IS*1246* (PP_2971), which are uniquely found on the KT2440 chromosome flanking *mrdH* and *mreA*, were found to be induced by the presence of Cd, Ni and Zn.

Additional putative heavy metal-responsive genes, many of which are absent in KT2440, F1 and GB-1 (Table 3), can be found on putative genomic islands on the W619 chromosome. A gene coding for a CDF putatively involved in zinc transport (PputW619_1714) and an additional copy of *arsB* (PputW619_1715) are located on region 8 (coordinates: 1875105–1910820). This region is located next to genes encoding proteins putatively involved in iron uptake (PputW619_1716-1719). On region 11 (coordinates: 2520458–2644620), two clusters of genes involved in mercury resistance (Fig. 4c) were found: a *merRB* (PputW619_2323-2324) locus, which contains a *merB* organomercurial lyase required for cleavage of mercury-alkyl bounds, and that is flanked by a copy of Tn*3* (PputW619_2321-2322) and an IS element from the IS*L3* family (IS*Ppu12*; PputW619_2325-2328); mercury resistance is completed by a *merRTPADE* operon (PputW619_2341-2336). This operon is flanked by a truncated transposase and a complete recombinase gene, and a copy of IS*Ps1*. This organization suggests the acquisition of mercury resistance via two events of horizontal gene transfer, followed by DNA rearrangements. On the putative genomic island region 18 (coordinates 3322597–3400760), a copy of *nikABCDE* (PputW619_3005-3009) encoding an ABC nickel uptake transporter, which was also found in KT2440, and a copy of *chrA* (PputW619_3017) involved in the transport of chromate were found. Additional heavy metal resistance or homeostasis genes were also identified outside putative genomic islands and include *cadAR* (PputW619_0325-0326), *modABC* (PputW619_3197-3199), *znuABC* (PputW619_5108-5111) and *cinAQ* (PputW619_1676-1677). These genes are common for all four *P. putida* strains. Among them, the copper-inducible genes *cinAQ* encode a copper-containing azurin-like protein and a pre-Q_0_ reductase, respectively, and are located adjacent to their two-component regulatory system *cinRS*. Gene disruptions of *cinA* and *cinQ* did not lead to a significant increase in the copper sensitivity of *P. putida* KT2440, which might result from the redundancy of copper resistance systems (Quaranta *et al.*, 2007). In addition, KT2440, F1 and GB-1 have a duplicated Cd/Zn/Co resistance system, *czcABC*, which is lacking in W619.

### Degradation of organic solvents and aromatic compounds by *P. putida*

Previous genome analysis of *P. putida* KT2440 revealed many metabolic pathways for the transformation of aromatic compounds (Jimenez *et al.*, 2002; Nelson *et al.*, 2002;). Putative genes coding for the degradation of aromatic compounds were compared among the four *P. putida* strains (see Table 4 and Table S5). Some of these aromatic compounds (ferulate, coumaryl alcohols, aldehydes and acids, *p*-hydroxybenzoate) may arise from the decomposition of plant materials, as can be found in the rhizosphere.

**Table 4:** Aromatic compound degradation pathway in *Pseudomonas putida* F1, and comparison with KT2440, W619 and GB-1

Gene	Product	F1 ORF ID Pput_	KT2440 ORF ID PP_	GB-1 ORF ID PputGB1_	W619 ORF ID PputW619_
*sepA/ttgA*	RND family efflux transporter	2867	1386	4427	1026
*sepB/ttgB*	Hydrophobe/amphiphile efflux	2868	1385	4428	1025
*sepC/ttgC*	RND efflux outer membrane lipoprotein	2869	1384	4429	1024
*todT*	Response regulator	2871	–	–	–
*todS*	Signal transduction histidine kinase	2872	–	–	–
*todE*	3-methylcatechol 2,3-dioxygenase	2876	–	–	–
*todD*	*cis*-Toluene dihydrodiol dehydrogenase	2877	–	–	–
*todA*	Aromatic-ring-hydroxylating dioxygenase	2878	–	–	–
*todB*	Ferredoxin	2879	–	–	–
*todC2*	Toluene dioxygenase	2880	–	–	–
*todC1*	Toluene dioxygenase	2881	–	–	–
*todF*	2-Hydroxy-6-oxo-2,4-heptadienoate hydrolase	2882	–	–	–
*todX*	Membrane protein	2883	–	–	–
*-*	Enoly-coenzyme A hydratase	2887	–	–	–
*mhpT*	3-Hydroxyphenylpropionic acid transporter	–	–	–	1985
*cmtG (mhpE)*	4-Hydroxy-2-oxovalerate aldolase	2888	–	–	19842007
*cmtH (mhpF)*	Acetaldehyde dehydrogenase	2889	–	–	19832008
*cmtF (mhpD)*	2-Hydroxypenta-2,4-dienoate hydratase	2890	–	–	19822011
*mhpC*	2-Hydroxy-6-ketonona-2,4-dienedioic acid hydrolase	–	–	–	1981
*mhpB*	2,3-Dihydroxyphenylpropionate 1,2-dioxygenase	–	–	–	1980
*mhpA*	3-(3-Hydroxy-phenyl)propionate hydroxylase	–	–	–	1979
*mhpR*	Mhp operon transcriptional activator	–	–	–	1978
*cmtE*	HOMODA hydrolase	2891	–	–	–
*cmtI*	Protein of unknown function	2892	–	–	–
*cmtD*	HCOMODA decarboxylase	2893	–	–	–
*cmtAd*	*p*-cumate dioxygenase ferredoxin subunit	2894	–	–	–
*cmtB*	2,3-dihydroxy-2,3-dihydro-*p*-cumate dehydrogenase	2895	–	–	–
*cmtC*	2,3-dihydroxy-*p*-cumate-3,4-dioxygenase	2896	–	–	–
*cmtAc*	*p*-Cumate dioxygenase small subunit	2897	–	–	–
*cmtAb*	*p*-Cumate dioxygenase large subunit	2898	–	–	–
*cmtAa*	*p*-Cumate dioxygenase ferredoxin reductase subunit	2899	–	–	–
*cymE*	Acetyl-coenzyme A synthetase	2900	–	–	–
*cymD*	Outer membrane protein	2901	–	–	–
*cymAb*	*p*-Cymene monooxygenase reductase subunit	2902	–	–	–
*cymAa*	*p*-Cymene monooxygenase	2903	–	–	–
*cymC*	*p*-Cumic aldehyde dehydrogenase	2904	–	–	–
*cymB*	*p*-Cumic alcohol dehydrogenase	2905	–	–	–
*cymR*	Regulatory protein for *cym* and *cmt* operons	2908	–	–	–

The gene annotations and comparisons were obtained using the MaGe annotation platform (Vallenet *et al.*, 2006).

Although KT2440 has versatile metabolic pathways to degrade aromatic compounds, it is not able to grow on any aromatic hydrocarbon as a sole carbon source. In contrast, with a significantly wider range of growth substrates, *P. putida* F1 is best known for its capability of growth on several aromatic hydrocarbons, including benzene, toluene, ethylbenzene and *p*-cymene. Its toluene degradation (*tod*) pathway is featured by the toluene dioxygenase operon *todABCDE* (Zylstra *et al.*, 1988; Gibson *et al.*, 1990;) and the corresponding two-component regulatory system, *todST* (Lau *et al.*, 1997). Besides the ability to use toluene, ethylbenzene or benzene as the sole carbon source, F1 is also able to grow on *p*-cymene (*p*-isopropyltoluene) and its acid derivative, *p*-cumate (Eaton, 1996, 1997). The mechanism uses two pathways: the *cymAaAbBCDER* pathway (Pput_2900-2905, 2908), responsible for the oxidation of *p*-cymene to *p*-cumate, and the adjacently located *cmtAaAbAcAdBCDEFGHI* pathway (Pput_2888-2899) to take *p*-cumate to isobutyrate, pyruvate and acetyl coenzyme A (Eaton, 1996, 1997). In F1, the *cym/cmt* and the *tod* pathways are located less than 3 kb apart on a putative genomic island (3260962–3302713), which is featured by a lack of synteny with other *P. putida* strains and surrounded by phage-related genes, an organization that is indicative that this region was acquired via transduction. A *sepABC* gene cluster is located upstream of this genomic island, encoding for solvent efflux or multidrug pumps (Phoenix *et al.*, 2003). KT2440, W619 and GB-1 all have conserved homologs for this cluster, referred to as *ttgABC* (Duque *et al.*, 2001). In addition, genomic sequence comparisons predicted the universal existence of other aromatic catabolic pathways in all four *P. putida* strains, including the protocatechuate (*pca* genes) and catechol (*cat* genes) branches of the β-ketoadipate pathway, and the phenylacetate pathway (*pha* genes) (see Table S5).

The genome of W619 contains two *mph* operons for the degradation of 3-hydroxyphenylpropionate (Ferrandez *et al.*, 1997): one complete *mhpRABCDFET* operon encoding enzymes for the conversion from 3-HTT to acetyl coenzyme A (CoA) and another incomplete cluster consisting of *mhpEFD*. The *mhpEFD* genes encode three enzymes that are conserved with part of the *cym/cmt* pathway: a 4-hydroxy-2-oxovalerate aldolase, an acetaldehyde dehydrogenase and a 2-hydroxypenta-2,4-dienoate hydratase (Table 4), which are respectively able to catalyze the conversion of 2-keto-4-pentenoate to 4-hydroxy-2-ketovalerate, to pyruvate and acetaldehyde and finally to acetyl CoA. This indicates that W619 has a broader potential for the degradation of aromatic compounds as compared with KT2440 and GB-1, but is less versatile than F1. For instance, unlike F1, *P. putida* W619 was unable to metabolize toluene or TCE, a property that could be complemented via acquisition of the pTOM plasmid of *Burkholderia cepacia* BU61 (Taghavi *et al.*, 2005; Weyens *et al.*, 2009;). The W619 *mhpRABCDFET* operon is flanked by a truncated copy of IS*1182* (PputW619_1976-1977) and an IS element from the IS*30* family (IS*1382*; PputW619_1990), which are absent in KT2440, GB-1 and F1. The presence of these mobile genetic elements points toward the acquisition of this catabolic region via horizontal gene transfer.

### Mn(II) oxidation by *P. putida*

In the environment, Mn cycles between the soluble reduced form Mn(II) and the insoluble oxidized forms Mn(III and IV) that can adsorb other trace metals from the environment and react as potent oxidizing agents. Thus, the Mn redox cycle has beneficial effects on the bioavailability and geochemical cycling of many essential or toxic elements (Tebo *et al.*, 2005). *Pseudomonas putida* GB-1 is a Mn(II)-oxidizing model bacterium (Corstjens *et al.*, 1992). Because Mn(III, IV) oxides are able to bind trace metals, this feature makes *P. putida* GB-1 a good candidate for bioremediation of heavy metal-contaminated sites. However, the mechanism for Mn(II) oxidation still remains to be elucidated. Several random transposon mutagenesis experiments led to the identification of genes important for Mn oxidation: the *ccm* operon coding for *c*-type cytochrome synthesis genes (Caspi *et al.*, 1998; de Vrind *et al.*, 1998;), the MCO *cumA* (Brouwers *et al.*, 1999) and genes encoding a general secretory pathway (de Vrind *et al.*, 2003). A recent paper showed that the MCO CumA is dispensable for Mn(II) oxidation. Instead, a two-component regulatory system (MnxS/R) was found to be essential for Mn(II) oxidation (Geszvain & Tebo, 2009). This is consistent with the fact that *cumA* is present in both Mn(II)-oxidizing and non-Mn(II)-oxidizing *Pseudomonas* strains (Francis & Tebo, 2001). Moreover, through transposon mutagenesis of other Mn(II) oxidases, such as *mnxG* (PputGB1_2447) and PputGB1_2665, Mn(II) oxidation was only slowed, but not lost in the mutant strains (Yamaguchi, 2009). These findings suggest that *P. putida* GB-1 may have multiple Mn(II) oxidases, with the expression of alternate enzymes dominating under different environmental conditions. Comparative genomics further indicates that genes related to Mn(II) oxidation in GB-1 have homologs in *P. putida* KT2440, W619 and F1, except that W619 lacks the homolog for PputGB1_2665 (Table S6). The presence of these genes points to the potential for Mn(II) oxidation by KT2440, F1 and W619. Also, a link between Mn(II) oxidation and pyoverdine siderophore synthesis was reported for *P. putida* MnB1 (Parker *et al.*, 2004). Thus, Mn(II) oxidation may influence the pyoverdine-mediated iron uptake by Mn(II)-oxidizing fluorescent *P. putida* strains.

### Response to oxidative stress by *P. putida*

*Pseudomonas putida* strains thrive in environments that are characterized by oxidative stress including the rhizosphere, soils and sediments containing reactive metal species generated during the Mn-redox cycle, or reactive intermediates generated during the oxidative breakdown of aromatic hydrocarbons. As part of their adaptation, *P. putida* strains possess various enzymes including catalases, peroxidases and superoxide dismutases for defense against reactive oxygen species (ROS) (including superoxide, hydroperoxyl radical and hydrogen peroxide species), nitric oxide and phytoalexins (Hammond-Kosack & Jones, 1996; Zeidler *et al.*, 2004;). Comparative genomic analysis shows that many of the enzymes involved in the oxidative stress response are common among the *P. putida* species (Table S7). For example, the W619 genome encodes three superoxide dismutases: SodA, a Mn superoxide dismutase (PputW619_4269), SodB, an Fe superoxide dismutase (PputW619_0981), and SodC, a Cu/Zn superoxide dismutase (PputW619_2485). The *sodC* gene is located on a putative genomic island (region 12) and it is absent from the other *P. putida* strains. *Pseudomonas putida* W619 is also predicted to contain five catalases, KatA (PputW619_4722), KatB (PputW619_2032), KatE (PputW619_5113), KatG (PputW619_2235) and one putative catalase (PputW619_2390); two alkyl hydroperoxide reductases, AhpF and AhpC (PputW619_3186-3187), four additional putative alkyl hydroperoxide reductases (one putative AhpC, PputW619_1113 and three having an AhpD domain, PputW619_3104, PputW619_3108, PputW619_3238); a chloroperoxidase (PputW619_1849); two thiol peroxidases (PputW619_2803 and PputW619_3977); and two putative glutathione peroxidases (PputW619_1244, PputW619_1483), a glutathione oxidoreductase (Gor, PputW619_3188) and a glutaredoxin (PputW619_3239). Among these enzymes, the KatB and AhpD domain proteins, whose genes are located on three genomic islands (regions 9, 19 and 20), are unique to *P. putida* W619. We also identified an organic hydroperoxide resistance protein (Ohr, PputW619_1469) located adjacent to its organic hydroperoxide resistance transcriptional regulator (OhrR, PputW619_1470). Moreover, *P. putida* W619 is likely able to detoxify free radical nitric oxide by the presence of a flavohemoprotein nitric oxide dioxygenase (PputW619_4378) with its anaerobic nitric oxide reductase transcription activator NorR (PputW619_4379) (Tucker *et al.*, 2004).

The oxidative stress response system is controlled via complex regulatory networks (Storz & Imlay, 1999). One of the key regulators is the peroxide resistance protein PerR, which was identified as the major regulator of the inducible peroxide stress response in *Bacillus subtilis* (Mongkolsuk & Helmann, 2002). In *P. putida* W619, the PerR protein is encoded by PputW619_2615, a LysR family transcriptional regulator that shares 61.5% identity with PerR of *E. coli* K12. PerR regulates the expression of *dps* (DNA-binding stress protein, PputW610_4005), *fur* (the ferric uptake repressor, PputW619_0702), *ahp*CF and *kat*A (Mongkolsuk & Helmann, 2002), all of which are present in *P. Putida* W619.

In some cases, the regulation of ROS-responsive genes is iron dependent and coupled to that of iron-binding proteins, such as bacterioferritin (Bfr). As in KT2440, the bacterioferritin α subunit (*bfr*A, PputW619_4721) in *P. putida* W619 is located next to catalase A (*kat*A) (Dos Santos *et al.*, 2004), and the *bfr*B and Bfd-associated ferredoxin gene (PputW619_1111-1112) are located adjacent to *ahp*C (PputW619_1113). Furthermore, various oxidation-resistant metabolic enzymes were putatively identified in W619, including *acn*A (encoding an aconitase, PputW619_1631), which is stable under oxidative stress, and *fum*C (PputW619_4271), located upstream of *sod*A (PputW619_4269), which encodes a hydroperoxide resistant isoform of fumarase. In *P. aeruginosa*, the *fum*C-*sod*A operon is shown to be negatively regulated in the presence of iron via the Fur protein (Polack *et al.*, 1996).

### Siderophore production by *P. putida*

The bioavailability of iron in the soil is limited by the very low solubility of the Fe^3+^ ion, and bacteria have developed diverse mechanisms for iron acquisition. One of the most important mechanisms is the production and release of siderophores to scavenge iron from the environment via the formation of soluble Fe^3+^ complexes, which can subsequently be taken up by active transport systems (Neilands, 1995; Ravel & Cornelis, 2003;). In addition to their own siderophores, bacteria may utilize a large number of heterologously produced siderophores that are taken up via various siderophore receptors (Dean *et al.*, 1996). Many *Pseudomonas* strains show fluorescence under UV light, indicative of the production of the siderophore pyoverdine (PVD) (Meyer, 2000). In accordance with this observation, nonribosomal peptide synthetase (NRPS) genes for pyoverdine synthesis were found in all four *P. putida* strains (Table S8). A cluster of peptide NRPS genes was identified, together with the PVD ABC transporter gene *pvdE* and a TonB-dependent receptor gene *fpvA*. At a separate locus, the chromosphore NRPS gene *psvA* was found to be present in all four strains along with the σ factor *pvdS* and, with the exception of strain F1, the siderophore biosynthesis gene *pvdZ* (Moon *et al.*, 2008). In F1, the *pvdZ* gene is located next to the *pvdE* gene. It should also be noted that the four *P. putida* strains are lacking the homologs of pseudomonine synthesis genes as identified in *P. entomopila* L48 (PSEEN_2500-2507), and therefore seem to be unable to produce this secondary siderophore (Matthijs *et al.*, 2009).

For the transport of pyoverdine, *P. putida* W619 possesses multiple copies (20) of genes encoding TonB-dependent outer-membrane receptors, as reported previously by Cornelis & Bodilis (2009). Out of these 20 TonB-dependent receptors, 17 are putative ferric siderophore receptors, including a putative ferric enterobactin receptor (*fepA*) and a FecA protein for ferric citrate uptake (Table S8). Of the three remaining TonB-dependent receptors, one is a putative copper-regulated channel protein (OprC) (PputW619_4630) (Yoneyama & Nakae, 1996), while the specificity of the other two receptors remains unclear (PputW619_1083, PputW619_5001). As summarized in Cornelis & Bodilis (2009), the genomes of F1, KT2440 and *P. entomophila* L48 carry 29 to 31 putative TonB-dependent receptor genes, a number considerably higher than the 20 genes identified in W619. It has been shown experimentally that *P. entomophila* L48 is able to utilize a large variety of heterologous pyoverdines, but that in contrast, KT2440 can only use its own pyoverdine and the pyoverdine heterologously produced by *P. syringae* LMG 1247 (Matthijs *et al.*, 2009). This observation is in accordance with the fact that L48 has the highest number of ‘orphan’ TonB-dependent receptor genes (11) that are not found on the genomes of the *P. putida* strains, while the *P. putida* KT2440, F1 and W619 genomes only contain 4, 3 and 5 ‘orphan’ genes, respectively. Thus, the capacity of W619 to utilize heterologous pyoverdine needs further investigation (Cornelis & Bodilis, 2009).

The TonB-dependent siderophore receptors genes in W619 are often located adjacent to genes coding a transmembrane anti-σ sensor (FecR family) and an ECF-σ70 transcriptional regulator of the FecI family, which might regulate the expression of receptors (Folschweiller *et al.*, 2000; Braun & Endriss, 2007; Cornelis *et al.*, 2009;). It should be noted that in the genomes of *P. putida* strains, up to 13 ECF-σ factors were found that showed similarity to the FecI-σ factor of *E. coli* (Martinez-Bueno *et al.*, 2002). The specificity of the receptors for various ferric siderophores is not well understood, but the presence of multiple TonB-dependent siderophore receptors might confer *P. putida* strains with the ability to use a broad spectrum of these heterologously synthesized compounds (Paulsen *et al.*, 2005). For example, the *P. putida* strains possess an iron uptake system that involves the genes coding for the outer-membrane ferric enterobactin receptor, PfeA/FepA, and its corresponding two-component regulator, PfeRS (Dean *et al.*, 1996). This system may allow *P. putida* to compete with *Enterobacteriaceae* for iron by utilizing the ferric–enterobactin complex. In addition, a universal TonB-dependent heme receptor gene (*phuR*) was found in all four *P. putida* strains (PP_1006, PputW619_4218, Pput_1043, PputGB1_1005), while two other heme uptake-related genes, *HasR* and *HxuC*, typically found in the pathogens *P. aeruginosa* (Ochsner *et al.*, 2000) and *P. entomophila* (Cornelis & Bodilis, 2009), were absent in *P. putida*. The redundant presence of systems that scavenge iron from the environment argues for the importance of iron acquisition in the ecology of this bacterium and its ability to colonize multiple niches.

## Association of *P. putida* with plants

Both *P. putida* KT2440 and W619 were found to live in association with plants, either as a rhizospheric strain or as an endophyte, respectively. Comparative genomics, using *P. putida* W619 as the reference strain, was used to identify the functions that are important for the association of these bacteria and their host plant.

### Motility

The *P. putida* W619 genome contains a large cluster of fifty-two genes involved in flagella biosynthesis (PputW619_3655-3737) (Table S9). This cluster is similarly organized in all four *P. putida* strains, except that W619 contains within the flagellar biosynthesis cluster a gene cluster involved in LPS biosynthesis and sporulation. Furthermore, the flagellar biosynthesis clusters in W619, KT2440 and F1 are interrupted by a region of atypical composition and lost synteny that contains d-Alanine and cystathionine ligases (genomic region 22; PputW619_3671-3677). *Pseudomonas putida* KT2440 is known to be a good swimmer; this in contrast to the solvent-tolerant *P. putida* DOT-T1E (Segura *et al.*, 2004). It was demonstrated that the impaired swimming capability of DOT-T1E resulted from a mutation in the *flhB* gene, which resulted in higher solvent resistance (Segura *et al.*, 2004). The *P. putida* W619 *flhB* gene is 91% identical to that of KT2440, suggesting a swimming capability similar to KT2440. In addition, it was demonstrated in KT2440 that mutations in the flagellar biosynthesis genes *flgL* (PputW619_3721), *fliA* (PputW619_3667), *fleQ* (PputW619_3698), *fliL* (PputW619_3691), *fliN* (PputW619_3683) and *flgD* (PputW619_3730) resulted in different degrees of impaired swimming motility, and swarming, and also affected the adhesion to seeds (Espinosa-Urgel *et al.*, 2000; Yousef-Coronado *et al.*, 2008;). The preliminary microarray result obtained with *P. putida* W619 showed that the transcription of genes encoding the flagellar proteins (PputW619_3724-3726) is induced when the cells are grown hydroponically in the presence of poplar roots, pointing toward a role of mobility in the plant–bacterial recognition/interaction process (data not shown).

### Pili and curli fibers

Type I pili assembly occurs in the periplasm and involves the chaperone–usher pili assembly system (Thanassi *et al.*, 1998). The four *P. putida* genomes all encoded a cluster of type I pili synthesis proteins, CsuABCDE, where CsuC and CsuD are predicted as the usher pathway chaperone and the usher protein, respectively (Table S10).

Type IV pili are typically 5-7 nm fibers that play a very important role in host colonization by a wide range of plant and animal pathogens (Mattick, 2002). The biogenesis and function of type IV pili are controlled by a large number of genes. On the *P. putida* genomes, three clusters of type IV pili biosynthesis genes were identified, *pil*MNPQ, *pil*ACD and *pil*EXW/*fim*T, as well as four copies of the *pil*Z gene for pili assembly. The pili assembly might be linked to the cell cycle, because *pil*Z is transcriptionally coupled to *hol*B, which encodes the δ subunit of DNA polymerase III (Alm *et al.*, 1996). Also *P. putida* contains a complex pili biosynthesis regulatory system, *pilGHIJLchpA*, where *pil*L/*chp*A encode a large fusion protein that acts as a very sophisticated signal transduction protein, ChpA (Alm & Mattick, 1997). Moreover, type IV pili are involved in natural DNA uptake (transformation). There are two additional putative *traX* genes (PputW619_2699, PputW619_5167) in *P. putida* W619, related to the conjugal DNA transfer that are absent in KT2440, F1 and GB-1.

Curli fibers are involved in adhesion to surfaces, cell aggregation and biofilm formation and play an important role in host cell adhesion and invasion (Barnhart & Chapman, 2006). The *P. putida* strains possess two gene clusters for curli fiber biogenesis: *csg*EFG and *csg*AB. The CsgAB proteins provide the two curli structural subunits, while CsgEFG are accessory proteins required for curli assembly (Hammar *et al.*, 1995).

### Colonization of seeds by *P. putida*

In *P. putida* KT2440, colonization of seeds was affected by mutations in various genes (Yousef-Coronado *et al.*, 2008). As expected, homologs of these genes were identified in *P. putida* W619: *lapA* (PputW619_5060), *lapBCD* (PputW619_5062-5064), *hemN2* (PputW619_0364), *coxE* (PputW619_0130), *galU* (PputW619_3189) and a hypothetical protein (PputW619_0162) that was shown to be involved in the oxidative stress response. We hypothesize that these genes have the same functions in W619. For instance, the LapA protein involved in biofilm formation is exported to the outside of the cell via the type I LapBC secretion system (Hinsa *et al.*, 2003). Furthermore, *P. putida* W619 contains an additional gene coding for an outer membrane component of the type I secretion system (PputW619_5061). The genome of *P. putida* W619 also encodes a putative adhesin (PputW619_3808) and a surface-adhesion calcium-binding outer membrane-like protein that was 24% identical to LapA. This large (3923 aa) protein is encoded next to its putative type I secretion system in a gene organization similar to the *lapABC* cluster. Other genes on the *P. putida* W619 genome putatively involved in adhesion include PputW619_1818 and PputW619_1489, both of which code for autotransporter proteins (secretion type V) with a pectin/lyase/pertactin domain. In contrast to the poplar endophyte *Enterobacter* sp. 638, no genes encoding a hemagglutinin protein were identified on the *P. putida* W619 genome. Among closely related *Pseudomonas* strains, we only found hemagglutinin-like adhesion genes in the insect pathogen *P. entomophila* L48 (PSEEN_0141, PSEEN_2177, PSEEN_3946), where they are predicted to be important virulence factors and involved in adhesion and type I or two-partner secretion systems (Vodovar *et al.*, 2006).

### Establishment of *P. putida* W619 in poplar

The *ndvB* gene (PputW619_2133) encodes a protein (2881 aa) involved in the production of β-(1,2)-glucan. The membrane-bound NdvB protein catalyzes three enzymatic activities: the initiation (protein glucosylation), elongation and cyclization in *situ* of β-(1,-2)-glucan, which is then released into the periplasm (Castro *et al.*, 1996). It has been reported that cyclic β-(1,2)-glucan is involved in the attachment of *Agrobacterium tumefaciens* to plant cells (Douglas *et al.*, 1985). In *Rhizobium meliloti*, mutations of the *ndvB* gene reduced the amounts of periplasmic β-(1,2)-glucan, which resulted in altered phenotypes related to phage and antibiotic sensitivity, motility, and growth in low-osmolarity media. Bacteroids produced by two of the downstream mutants were morphologically abnormal, indicating that *ndvB* is involved not only in invasion but also in bacteroid development (Ielpi *et al.*, 1990). The *ndvB* gene is not present in *P. putida* GB-1, F1 or KT2440, but was identified in the other two poplar endophytes: *Serratia proteamaculans* 568 and *Enterobacter* sp. 638.

## Plant growth-promoting properties of *P. putida*

### Synthesis of plant growth-promoting hormones by *P. putida*

#### Indole-3-acetic acid

Many plant growth-promoting bacteria are capable of synthesizing phytohormones that affect the growth and development of their plant host. *In vitro, P. putida* W619 was the most efficient producer of IAA in comparison with other endophytic bacteria (Taghavi *et al.*, 2009). Consistently, the *P. putida* W619 genome encodes two putative tryptophan-dependent IAA synthesis pathways. One is via tryptamine and indole-3-acetaldehyde and requires a tryptophan decarboxylase (PputW619_2223), an amine oxidase (PputW619_0482) and an indole-3-acetaldehyde dehydrogenase (encoded by many putative genes PputW619_0192/0213/0597/2257/2639/2872/2926/2546/3767). This pathway also exists in KT2440, F1 and GB-1. The alternate pathway is converting tryptophan to indole-3-acetamide via a tryptophan 2-monooxygenase (PputW619_1175/4820), which is subsequently converted into IAA by a putative deaminase (PputW619_2551). *Pseudomonas putida* KT2440, F1 and GB-1 lack homologs for PputW619_1175, but contain conserved genes for PputW619_4820. Furthermore, the step to convert indole-3-acetamide into IAA seems to be absent in KT2440; thus, this pathway might be incomplete and nonfunctional. Along with the high level of IAA production, *P. putida* W619 has three genes encoding putative auxin efflux carriers (PputW619_2484/2492/3829). *Pseudomonas putida* KT2440, F1 and GB-1 also have auxin efflux carriers, but only in two copies for F1 and KT2440.

#### ACC deaminase

A functional 1-aminocyclopropane-1-carboxylate deaminase (*acd*) (EC: 3.5.99.7), involved in counteracting the plant's ethylene stress response, is absent on the genomes of *P. putida* W619, GB-1, F1 and KT2440, as was confirmed by the failure of these strains to grow on 1-aminocyclopropane-1-carboxylate as the sole carbon or nitrogen source. Although putative ACC deaminases were identified, all lack the particular amino acids E296 and L323 (respectively, replaced by a T or S and a T) that constitute part of the signature sequence of the active site of the true ACC deaminases and are involved in substrate specificity (Glick *et al.*, 2007).

#### Acetoin and 2,3-butanediol synthesis

Volatile compounds such as 3-hydroxy-2-butanone (acetoin) and 2,3-butanediol are emitted by rhizobacteria to enhance plant growth (Ryu *et al.*, 2003). In contrast to the poplar endophyte *Enterobacter* sp. 638, whose genome was sequenced recently (Taghavi *et al.*, 2010), the *P. putida* W619 genome does not encode the *budABC* operon involved in the conversion of pyruvate to acetoin and 2,3-butanediol. The *P. putida* W619 genome carries the gene *poxB* (PputW619_2979) encoding a pyruvate dehydrogenase (genomic region 18). While the principal function of this enzyme is to convert pyruvate into acetaldehyde, a small fraction of the pyruvate can be converted into acetoin as a byproduct of the hydroxyethyl-thiamin diphosphate reaction intermediate. Although the production of acetoin by *P. putida* W619 is probably very low, especially at a low pyruvate concentration, this plant growth hormone can be utilized and converted into 2,3-butanediol, another plant growth hormone, by the poplar tree.

On the genomes of *P. putida* F1 (Pput_0595-0591), GB-1 (PputGB1_0601-0597) and KT2440 (PP_0556-0552) genes (*acoXABC adh*) involved in the catabolic conversion of acetoin and 2,3-butanediol to central metabolites were identified. Interestingly, these genes were not present in *P. putida* W619. Therefore, *P. putida* W619 does not have an antagonist effect on the production of the plant growth-promoting phytohormones acetoin and 2,3-butanediol, which might be beneficial as these compounds stimulate root formation and indirectly the availability of carbon sources for KT2440 when residing in the plant rhizosphere.

#### Salicilic acid

The genome of *P. putida* W619 encodes a salicylate 1-monooxygenase (*mahG*, PputW619_2140) involved in the conversion of salicylate into catechol. Homologues of this gene were also found on the genomes of *P. putida* KT2440, F1 and GB-1. Salicylate is a phenolic phytohormone involved in plant growth and development, and plays a role in the resistance to pathogens by inducing the production of pathogenesis-related proteins (Van Huijsduijnen *et al.*, 1986).

### Defense against plant pathogens by *P. putida*

Many pathogenic fungi secrete mannitol, a powerful reactive oxygen scavenger, into the apoplast during plant infection (Jennings, 1984; Velez *et al.*, 2007;). This process has been shown to be required for pathogenicity as it suppresses the reactive oxygen-mediated plant host defenses (Chaturvedi *et al.*, 1996a,b; Velez *et al.*, 2008;). The production of mannitol dehydrogenase (MTD, EC 1.1.1.255) was hypothesized to increase the effectiveness of the plant defense response via the conversion of pathogen-secreted mannitol into mannose (Jennings *et al.*, 2002). Interestingly, the gene encoding for MTD was found in *P. putida* W619 (*mltD*, PputW619_2037) located on a putative genomic island (region 9), along with genes coding for a mannitol ABC transporter (*mltKGFE*, PputW619_2038-2041), a transcriptional activator (*mltR*, PputW619_2042), a fructokinase (*mltZ*, PputW619_2035) and a xylulokinase (*mltY*, PputW619_2036). It is therefore hypothesized that *P. putida* W619 can assist its plant host in its defense against pathogenic fungi, an important property for its commensal life style. The *mltZYDKGFE* cluster, which is very similar to the operon found in *P. fluorescens* DSM50106 (Brunker *et al.*, 1998a,b;), is absent in *P. putida* KT2440, F1 and GB-1.

## Virulence factors in *P. putida*

Like for the granted biosafety strain *P. putida* KT2440, strains W619, F1 and GB-1 also lack a number of key determinants required for virulence and virulence-associated traits (Nelson *et al.*, 2002). There is no evidence in the four *P. putida* genome sequences for putative functions required for the biosynthesis of exotoxin A, phospholipase C or pectin lyase that are frequently present in plant and animal pathogens. Additionally, the type III secretion pathway present in the plant pathogens *P. syringae* pv. *tomato* DC3000 or pv. *phaseolicola* 1448A (Cornelis & Van Gijsegem, 2000; Feil *et al.*, 2005; Vencato *et al.*, 2006;) is missing in all four *P. putida* strains. Some individual homologs with putative functions were found, such as PputW619_0806 coding for a putative type III HopPmaJ effector. However, this is in sharp contrast to the presence of a 20-kb gene cluster encoding a putative type III secretion system in the rhizosphere bacterium *P. fluorescens* SBW25 (Preston *et al.*, 2001). Components of the type VI secretion system (T6SS) were also identified in *P. putida* strains and compared with the three T6SS clusters described in *P. aeruginosa* (Filloux *et al.*, 2008) (Table S11). The T6SS clusters are partially conserved among the *P. putida* strains, including the ATPase ClpV, and the secreted VgrG and Hcp proteins. In W619, one of the T6SS-associated gene clusters (PputW619_3242-43, 3245-46, 3255-56, 3260-61) is located in genomic region 20 (3574758–3603632). Although the majority of genomic region 20 appears to lack synteny with the other three *P. putida* strains, the T6SS-associated genes seem to be conserved among KT2440, F1 and GB-1. Furthermore, in region 20, GAF/GGDEF and EAL signaling domains (Galperin, 2004) were identified that are hypothesized to play a role in regulating bacteria–host interactions. It also needs to be examined how far the functions of the putative T6SS in *P. putida* differ from the systems described in other Pseudomonads. For instance, they do not seem to play a role in virulence, as they lack homologs of the serine–threonine protein kinase (STPK), a key virulence factor in *P. aeruginosa* (Bingle *et al.*, 2008).

## Concluding remarks

The present inventory of genes and operons and the comparative genome analysis of the genome sequences of *P. putida* KT2440, W619, F1 and GB-1 provided a powerful tool to gain new insights into the adaptation of this species to specific lifestyles and environmental niches. Although *P. putida* strains are generally known for their versatile metabolic properties, this comparison also clearly demonstrated that horizontal gene transfer played an important role in this adaption process, as many of the niche-specific functions were found to be encoded on clearly defined genomic islands or on regions with lost synteny between the closely related strains. Often the genomic islands are delineated by mobile elements, many of which seem to be strain specific. This further supports the notion that these genomic islands were acquired rather than being part of the *P. putida* core genome.

The presence of many incomplete pathways as well as the duplication of pathways on the genomes of all four *P. putida* strains is striking. Examples include pathways involved in heavy metal resistances such as the copper resistance systems in *P. putida* W619 or the duplication of *czc*CBA in *P. putida* F1, KT2440 and GB-1, the breakdown of aromatic compounds, such as the presence of a complete plus an incomplete *mph* operon for the degradation of 3-hydroxyphenylpropionate (3-HPP) in W619, or the presence of two alternative pathways for the synthesis of IAA in *P. putida* W619 compared with only one pathway in the other three strains. The presence of various complete and incomplete pathways indicates that these strains, before adapting to their current biotopes, have a rich history of colonizing various biotopes, each of which required specific adaptations that were facilitated via the acquisition and reshuffling of new pathways. These adaptations also required the dedicated management of incoming systems to avoid functional redundancy (i.e. under stress when resources are scarce), their optimization and the economical interplay between various pathways genes, supported by a large diversity of regulators typically found in *P. putida*. A similar duplication and interplay of functions was described recently for the adaptation of *C. metallidurans* CH34 to environments polluted by heavy metals (Janssen *et al.*, 2010). Whole-genome expression studies combined with metabolite analysis and proteomics should help to better understand the mechanisms for the interplay and roles of functions essential for the niche adaptation of these strains, and when combined with genome-scale metabolic reconstruction models (Nogales *et al.*, 2008; Puchalka *et al.*, 2008;), will provide a nonempirical basis for the development of biotechnological applications that can be further tailored as a function of strain-specific metabolic pathways.

As expected from the unique features of the niches from which the strains were isolated, *P. putida* F1 was the best adapted to degrade a variety of aromatic compounds, while W619 was more competent with regard to heavy metal resistances and beneficial effects on plant. A comparison between *P. putida* W619 and KT2440 is of special interest, as it could provide valuable clues to identify key functions for an endophytic lifestyle in comparison with survival in the rhizosphere. Noticeable differences include the presence of the *ndvB* gene in W619 involved in the production of β-(1,2)-glucan, a putative adhesin, a surface-adhesion calcium-binding outer membrane-like protein that was 24% identical to LapA and two genes putatively involved in adhesion that code for autotransporter proteins (secretion type V) with a pectin/lyase/pertactin domain. In addition, *P. putida* W619 seems to be a better producer of the plant growth-promoting compound IAA. However, as this inventory mainly relies on the comparison of the two strains for already known genes and mechanisms in plant-colonizing bacteria, genuine novel genes or functions may be overlooked. A combination of transcriptomics, proteomics, metabolomics and mutagenesis to study the plant colonization process will be of invaluable help in this respect: it will allow assigning new functions to putative genes and pathways, help to detect new proteins and confirm the metabolic potential of the strains. A similar approach is also required to better understand the mechanisms underlying the Mn(II)-oxidization ability of the various *P. putida* strains, because comparative genomics indicated that genes related to Mn(II) oxidation in GB-1 have homologs in *P. putida* KT2440, W619 and F1.

## Statement

Re-use of this article is permitted in accordance with the Terms and Conditions set out at http://wileyonlinelibrary.com/onlineopen#OnlineOpen_Terms

## Supporting Information

**Table S1.** Putative genomic islands identified on the chromosomes of *Pseudomonas putida* W619, KT2440, F1 and GB-1.

**Table S2.** Putative orthologous relationships between the chromosomes of the four *Pseudomonas putida* strains.

**Table S3.** Comparative analysis of the functional group distribution among bacteria, *Gammaproteobacteria, Pseudomonas*, and the four *P. putida* strains (KT2440, F1, GB-1 and W619), based on Clusters of Orthologous Groups of proteins (COGs) classification.

**Table S4.** Incomplete or truncated remnants of IS elements identified among the genomes of *Pseudomonas putida* KT2440,W619, F1 and GB-1.

**Table S5.** Complete overview of putative genes involved in the degradation of aromatic compounds identified on the genomes of *Pseudomonas putida* W619, KT2440, F1 and GB-1.

**Table S6.** Putative genes involved in Mn(II) oxidation identified on the genomes of *Pseudomonas putida* W619, KT2440, F1 and GB-1.

**Table S7.** Putative genes involved in the oxidative stress responce in *Pseudomonas putida* W619, KT2440, F1 and GB-1.

**Table S8.** Putative genes involved in siderophore synthesis and iron uptake.

**Table S9.** Putative genes involved in the motility of *Pseudomonas putida* strains.

**Table S10.** Putative genes involved in the pili and curli fibers biosynthesis.

**Table S11.** Conserved genes of the type VI secretion system of *Pseudomonas putida* strains compared with the three T6SS clusters identified in *Pseudomonas aeruginosa*.
